# An Antibody-Drug Conjugate That Selectively Targets Human Monocyte Progenitors for Anti-Cancer Therapy

**DOI:** 10.3389/fimmu.2021.618081

**Published:** 2021-02-22

**Authors:** Yuta Izumi, Masashi Kanayama, Zhongchuzi Shen, Masayuki Kai, Shunsuke Kawamura, Megumi Akiyama, Masahide Yamamoto, Toshikage Nagao, Keigo Okada, Norihiko Kawamata, Shigeo Toyota, Toshiaki Ohteki

**Affiliations:** ^1^ Department of Biodefense Research, Medical Research Institute, Tokyo Medical and Dental University (TMDU), Tokyo, Japan; ^2^ Oncology Research Laboratories, Oncology R&D Unit, R&D Division, Kyowa Kirin Co., Ltd., Tokyo, Japan; ^3^ Department of Hematology, Tokyo Medical and Dental University (TMDU), Tokyo, Japan; ^4^ Department of Hematology, Yokosuka Kyosai Hospital, Kanagawa, Japan

**Keywords:** monocyte, leukemia, tumor-associated macrophage, chronic myelomonocytic leukemia, common monocyte progenitor

## Abstract

As hematopoietic progenitors supply a large number of blood cells, therapeutic strategies targeting hematopoietic progenitors are potentially beneficial to eliminate unwanted blood cells, such as leukemic cells and immune cells causing diseases. However, due to their pluripotency, targeting those cells may impair the production of multiple cell lineages, leading to serious side effects such as anemia and increased susceptibility to infection. To minimize those side effects, it is important to identify monopotent progenitors that give rise to a particular cell lineage. Monocytes and monocyte-derived macrophages play important roles in the development of inflammatory diseases and tumors. Recently, we identified human monocyte-restricted progenitors, namely, common monocyte progenitors and pre-monocytes, both of which express high levels of CD64, a well-known monocyte marker. Here, we introduce a dimeric pyrrolobenzodiazepine (dPBD)-conjugated anti-CD64 antibody (anti-CD64-dPBD) that selectively induces the apoptosis of proliferating human monocyte-restricted progenitors but not non-proliferating mature monocytes. Treatment with anti-CD64-dPBD did not affect other types of hematopoietic cells including hematopoietic stem and progenitor cells, neutrophils, lymphocytes and platelets, suggesting that its off-target effects are negligible. In line with these findings, treatment with anti-CD64-dPBD directly killed proliferating monocytic leukemia cells and prevented monocytic leukemia cell generation from bone marrow progenitors of chronic myelomonocytic leukemia patients in a patient-derived xenograft model. Furthermore, by depleting the source of monocytes, treatment with anti-CD64-dPBD ultimately eliminated tumor-associated macrophages and significantly reduced tumor size in humanized mice bearing solid tumors. Given the selective action of anti-CD64-dPBD on proliferating monocyte progenitors and monocytic leukemia cells, it should be a promising tool to target cancers and other monocyte-related inflammatory disorders with minimal side effects on other cell lineages.

## Introduction

Chronic myelomonocytic leukemia (CMML), a hematopoietic malignancy characterized by the overproduction of monocytes and their progenitors, develops from genetic mutations in hematopoietic stem and progenitor cells (HSPCs) (1–3). CMML is classified as myelodysplastic syndrome/myeloproliferative neoplasm (MDS/MPN) (1, 4). Patients with CMML show excessive monopoiesis, dysplasia and inefficient hematopoiesis (1, 4), which often causes anemia, thrombocytopenia and infectious diseases (5). HSC transplantation is the only curative treatment for CMML patients (1, 2). However, HSC transplantation requires highly invasive pre-conditioning, carries risks of graft-versus-host disease (GVHD) and increased susceptibility to infection, which is not always applicable especially for elderly patients (the median age at the time of CMML diagnosis is 75 years old) (1). Instead, various agents have been used to control tumor burden and induce remission, but ineffectiveness (non-responders) and fatal myelosuppression remain as serious problems, causing a poor prognosis for CMML patients with 1–3 years of median overall survival (1, 6). Thus, to achieve an effective anti-leukemia therapy without disturbing normal hematopoiesis, agents with a high specificity against target leukemic cells are urgently required.

Recently, a class of molecular targeted agents named antibody-drug conjugates (ADCs) has been actively developed to avoid nonspecific cell elimination and to minimize collateral damage (7, 8). ADCs are composed of cytotoxic drugs (payload), linkers and a specific antibody (Ab), which enables the delivery of potent payloads to specific cells. In this context, the specificity for the killing activity of ADCs solely depends on the selection of a target molecule. To date, gemtuzumab ozogamicin (GO), an anti-CD33 ADC, was approved for treatment of acute myeloid leukemia (AML) by the Food and Drug Administration (FDA) in 2000 (2, 9), but to date no ADC has been approved for CMML therapy. However, CD33 is broadly expressed on normal myeloid progenitors as well as on leukemic cells of AML patients (10, 11), which causes severe myelosuppression, resulting in the voluntary withdrawal of GO from the US market in 2010 (12). GO was re-approved in 2017 and those adverse events revealed the importance of target molecule selection for ADC development. In this context, we recently reported that conventional granulocyte-monocyte progenitors (cGMP, Lin-CD34^+^CD38^+^CD10^-^CD123^low^FLT3^+^CD45RA^+^) are heterogeneous and contain common monocyte progenitors (cMoPs) that strictly give rise to monocytes (13). Based on that finding, we proposed a human monocyte differentiation pathway (13). Accordingly, targeting the human monocyte differentiation pathway including cMoPs could be beneficial for the therapy of CMML/AML because the progenitors can expand and generate large numbers of monocytic leukemia cells.

Monocytes and macrophages are harmful participants in some human solid tumors (14). For instance, TIE2-expressing monocytes (TEMs) enhance angiogenesis and tumor progression (15, 16) and macrophages in solid tumors, referred to as tumor-associated macrophages (TAMs), are a poor prognostic factor and exert multiple pro-tumorigenic effects such as the promotion of angiogenesis, extracellular matrix remodeling and immunosuppression (17–19). Based on that background, several drugs controlling TAMs have been developed (20–23). Colony stimulating factor 1 receptor (CSF1R) is a representative target for anti-TAM therapy because of its crucial roles in the generation and function of TAMs (24–26). However, comprehensive gene expression analysis revealed that a CSF1R blockade-resistant TAM subset likely exists in solid human tumors (18). Thus, TAMs have a remarkable plasticity and alter their function and surface molecule expression, which causes difficulties in therapeutically targeting them (27, 28).

In light of those considerations, we developed a unique ADC that selectively targets proliferating monocytic progenitors and monocytic leukemia progenitors but not mature monocytes and monocyte-derived macrophages. By blocking the sources of those cells, we succeeded in reducing leukemic monocytes in a patient-derived xenograft (PDX) model of CMML, in depleting TAMs and inversely inducing tumor regression in a solid tumor model. Importantly, the ADC treatment showed minimal cytotoxicity against multipotent HSPCs and other hematopoietic lineages. Consequently, the ADC targeting monocytic progenitors may serve as a therapeutic agent for monocytic leukemias, solid tumors and presumably monocyte-related inflammatory diseases.

## Materials and Methods

### Mice and Human Samples

NOG (*NOD/Shi-scid, IL-2RγKO Jic*) and human interleukin-6 transgenic NOG (hIL-6 Tg NOG) mice (29) were purchased from CLEA Japan. All mice were maintained under specific pathogen-free conditions. Human umbilical cord blood (UCB) samples were provided by the Japanese Red Cross Kanto-Koshinetsu Cord Blood Bank, and bone marrow (BM) samples from patients with CMML were provided by the Department of Hematology, Tokyo Medical and Dental University and the Department of Hematology, Yokosuka Kyosai Hospital under agreement of the patients. Human blood samples were donated by healthy volunteers. All experiments with human materials were approved by the Scientific Ethics Committees of the Japanese Red Cross Kanto-Koshinetsu Cord Blood Bank, the Medical Research Institute, the Tokyo Medical and Dental University and/or the Yokosuka Kyosai Hospital. Informed consent was obtained from all subjects.

### Antibodies and Reagents

LEGENDScreen Human PE Kit, mouse IgG1 isotype control antibody (MOPC-21), Brilliant Violet 711-conjugated streptavidin and anti-human antibodies against CD38 (HIT2), CD34 (581), CD10 (HI10a), CD123 (6H6), CD45RA (HI100), CD135 (BV10A4H2), CD64 (10.1), CLEC12A (50C1), CD45 (H130), CD14 (M5E2), CD16 (3G8), CD49f (GoH3), CD206 (15-2), CD19 (HIB19), CD56 (MEM-188, HCD56), CD4 (RPA-T4), CD8α (RPA-T8), CD11c (3.9), CD2 (RPA-2.10), CD3 (UCHT1), CD11b (ICRF44) and CD235ab (HIR2) were purchased from BioLegend. Anti-human antibodies against CD90 (eBio5E10), CD3 (UCHT1), HLA-DR (L243), CD66b (G10F5), CD41a (HIP8) and CD42b (HIP1) and anti-mouse TER-119 (TER-119) were purchased from eBioscience. Anti-human CD163 (REA812), SIGLEC-7 (REA214), SIGLEC-9 (REA492) and REA control (REA293) Abs were purchased from Miltenyi Biotec. The anti-human CD14 Ab (RMO52) was purchased from Beckman Coulter. Propidium iodide (PI) and phorbol 12-myristate 13-acetate (PMA) were purchased from Sigma-Aldrich. The Diff-quik staining kit was purchased from Sysmex Corp. To purify antibodies against human CD64 [clones H22 (30), 32.2 (31) and 611 (32)] and the isotype control Ab against dinitrophenol (DNP), expression vectors encoding the genes for IgG1 and an IgG4 analogue, called the nullbody, were synthesized with the S239C mutation, a site used to conjugate the payload (33, 34) and were transfected into Expi293 cells using an ExpiFectamine 293 Transfection Kit (Gibco). Antibodies in cell culture supernatants were obtained by affinity purification.

### Cell Lines

THP-1, U937 and Ramos cells were obtained from an in-house cell bank. The cells were cultured in Iscove’s Modified Dulbecco’s Media (IMDM) (Sigma-Aldrich) supplemented with 10% fetal bovine serum (FBS), 1% penicillin/streptomycin and 1% L-alanyl-L-glutamine (Wako) at 37°C in a 5% CO_2_ atmosphere. Human HSC4 head and neck squamous cell carcinoma cells were purchased from the Japanese Collection of Research Bioresources (JCRB) Cell Bank and were cultured in RPMI-1640 (Sigma-Aldrich) containing 20% FBS, 1% penicillin/streptomycin, 1% MEM NEAA (Gibco), 1% sodium pyruvate (Gibco), and 0.1% 2-mercaptoethanol (Gibco).

### Generation of ADC

The antibodies were reduced using Tris(2-carboxyethyl)phosphine hydrochloride (TCEP)-HCl and the reaction was carried out at 37°C for 1 h in PBS. Unreacted TCEP was then removed and replaced with oxidation buffer (20 mM phosphate, 150 mM NaCl, 1 mM EDTA, pH 6.5) using an Amicon Ultra-4 centrifuge device (Millipore). Next, (L)-dehydroascorbic acid (DHAA) was added to the antibodies to restore cross-linking between cysteines other than the S239C residue, and the oxidation reaction was carried out for 1 h at room temperature. After the reaction, unreacted DHAA was removed and replaced with conjugation buffer (1 mM EDTA, PBS) using an Amicon Ultra-4 centrifuge device. Propylene glycol was added to the antibodies and maleimide-va-dPBD (purchased from XDCExplorer) and the conjugation reaction was allowed to proceed by mixing them together. After the reaction, the unreacted maleimide-va-dPBD was removed and replaced with PBS using a NAP column (GE Healthcare). In addition, the resulting complexes were concentrated using an Amicon Ultra-4 centrifuge device and then filtered by an Ultrafree-centrifugal filter (Millipore).

### Cell Preparation From Human Samples

BM samples were obtained from CMML patients (**Supplementary Table 1**). Human mononuclear cells (MNCs) were collected from blood, BM, and UCB samples by density gradient centrifugation using Lymphocyte Separation Solution (*d* = 1.077, Nacalai Tesque). Regarding MNCs isolated from BM and UCB, cells were stained with PE/Cy5-conjugated Lineage Abs (Abs against CD2, CD3, CD11b, CD16, CD19, CD56, CD235ab, and CD14). After washing, cells were reacted with anti-Cy5-MicroBeads (Miltenyi Biotech) and lineage^+^ cells were depleted with an autoMACS Pro Separator (Miltenyi Biotech). After staining with specific antibodies and PI, cells were analyzed and/or sorted using a FACS Aria III or FACS Canto II (BD).

### Humanization of Immunodeficient Mice

NOG or hIL-6 Tg NOG mice were sublethally irradiated (1-2 Gy) and 1×10^5^ Lin^-^CD34^+^ cells from UCB were intravenously injected within 24 h after irradiation. To generate the PDX model, sublethally irradiated NOG mice received an intravenous injection of total MNCs or 2-3×10^5^ Lin^-^CD34^+^ cells isolated from BM samples of CMML patients. Two PDX mice were generated from each BM sample. To monitor their chimerism, circulating blood was collected every 4 weeks and the cells were analyzed by flow cytometry (FCM). After more than 8 weeks post-transplantation, 0.5 µg DNP-dPBD or H22-dPBD was intravenously administered once. At day 7 after the administration, the mice were sacrificed and analyzed.

### Generation of Tumor-Bearing Humanized Mice

HSC4 cells (1.5×10^6^ in 100 µl PBS) were subcutaneously injected into humanized hIL-6 Tg NOG mice 2–3 months after humanization. From day 7 after the transplantation, tumor sizes were measured every 4 days and treatments with 0.5 µg DNP-dPBD or H22-dPBD were performed at days 7, 14, and 21. MNCs in the blood, BM and tumors of mice were analyzed 28 days post-tumor inoculation.

### FCM Analysis of Humanized Mice

BM cells were obtained from the hind limbs of humanized mice by flushing with PBS containing 0.5% bovine serum albumin. BM cells and blood cells were hemolyzed and stained with specific antibodies. For preparation of tumor-associated cells, tumor tissues were minced and digested in RPMI-1640 medium containing collagenase (0.5 mg/ml, Sigma-Aldrich) and DNase I (5 µg/ml, Roche) for 30 min at 37°C. After digestion, cells were filtered through a 100 µm cell strainer. Tumor-associated leukocytes were isolated by density centrifugation with Percoll (Cytiva) and then analyzed using a FACS Aria III or FACS Canto II (BD) after staining with antibodies and PI.

### Internalization Assay

To examine internalization and colocalization of the H22 nullbody in lysosomes, sorted CD14^hi^CD16^-^ monocytes were stained with Alexa Fluor 647-labeled H22 nullbody. The cells were then washed with culture media and cultured at 37°C for 23 h. After culture, cells were incubated with 100 nM LysoTracker Red DND-99 (Invitrogen) for 1 h. After washing and fixation with 4% paraformaldehyde (PFA) in PBS (Nacalai tesque), cell images were obtained using a TCS SP8 confocal microscope (Leica). For the quenching assay, U937 cells (5×10^4^/well) were plated in 96-well U-bottom plates. After adding the Alexa Fluor 488-labeled H22 nullbody (50 μg/ml), cells were incubated on ice for 1 h or at 37°C for 1, 4, 8 or 24 h. After washing, cells were treated with 100 nM quenching Ab (anti-Alexa Fluor 488 Rabbit IgG, Invitrogen) on ice for 1 h. After washing with PBS containing 0.1% BSA and 0.1% NaN_3_, signals from the internalized Alexa Fluor 488-labeled Ab were detected by FCM.

### Competitive Inhibition Assay for Anti-CD64 Antibodies

THP-1 cells (2×10^5^ per well in 96-well plates) were treated with purified IgG from human serum (Miltenyi Biotec) at 4°C for 10 min. After incubation with non-labeled nullbodies (10 μg/ml) at room temperature for 30 min, Alexa Fluor 647-labeled nullbodies (0.1 μg/ml) were added and incubated at room temperature for 30 min. After washing, cells were stained with 7-AAD and signals of Alexa Fluor 647 were detected by FCM.

### *In Vitro* Cytotoxicity Assay

Cells were cultured with DNP-MMAE, H22-MMAE, DNP-dPBD, or H22-dPBD in the presence or absence of human serum (Sigma) for 4 or 6 days and cell viabilities were assessed using a CellTiter-Glo Luminescent Cell Viability Assay (Promega) according to the manufacturer’s instructions. To test the impact of ADC-treatment on monocyte production from myeloid progenitors, Lin^-^CLEC12A^+^ cells (for CellTiter-Glo analysis, 3×10^3^ cells/well in 96-well plates; for FCM analysis, 1×10^4^ cells/well in 96-well plates) were cultured in IMDM supplemented with 10% FBS, 1% penicillin/streptomycin, 1% L-alanyl-L-glutamine, 100 ng/ml stem cell factor (SCF), 50 ng/ml thrombopoietin (TPO) and fms-like tyrosine kinase receptor-3 ligand (FLT3L) in the presence or absence of H22-dPBD or DNP-dPBD for 6 days. In some experiments, THP-1 cells were irradiated at 5 Gy immediately before the ADC-treatment, treated with 40 ng/ml PMA 24 h prior to the ADC-treatment or cultured with the IMDM medium containing 1% or 2% FBS during the ADC-treatment. To examine the antigen specificity of H22-dPBD, THP-1 cells were treated with H22-dPBD (0.04 μg/ml) in the presence of unconjugated H22 antibody (4 μg/ml). The viability of THP-1 cells was examined using a CellTiter-Glo Luminescent Cell Viability Assay at day 6. For the quantification of apoptotic THP-1 cells, the cells were cultured in the presence of 0.04 μg/ml DNP-dPBD or H22-dPBD for 3 days, and they were stained with PE-conjugated annexin V (BD Bioscience) and analyzed using a FACS Canto II.

### THP-1 Xenografts

NOG mice were intravenously injected with 4×10^5^ THP-1 cells after sublethal irradiation (1-2 Gy). The mice were intravenously treated with 0.5 μg ADCs at day 14 after xenografting and were analyzed by FCM at day 21. When the survival of mice was examined, mice received ADC-treatments at days 14 and 28 after xenografting and their survival was monitored until day 56.

### Immunofluorescence Staining of Frozen Tumor Sections

HSC4 tumor tissues obtained from hIL-6 Tg NOG mice were embedded in O.C.T. Compound (Sakura Finetek Japan) by liquid nitrogen. The samples were sliced into 8 µm sections and placed on slides. After washing in PBS and fixation by 4% PFA (10 min at room temperature), the sections were blocked with a two-fold dilution of Block ACE (Bio-Rad) for 30 min. Immunostaining was performed for 1 h at room temperature with a 50-fold dilution of APC-conjugated anti-human CD163 Ab (REA812) or isotype-matched control Ab in the presence of a 10-fold dilution of Block ACE. The sections were further stained with 5 µM DAPI for 10 min and images were obtained using a BZ-X710 (Keyence).

### Statistical Analysis

Statistical analyses were performed using Prism software version 7 (GraphPad). A two-tailed Student’s *t*-test or multiple *t*-test was used for statistical analyses of two-group comparisons. Multigroup comparisons were performed by a one-way analysis of variance (ANOVA) followed by the Tukey–Kramer multiple comparisons test. For statistical evaluation of survival, the Gehan-Breslow-Wilcoxon test was used. The criterion of significance was set at *p* < 0.05. All results are expressed as means ± standard deviation of the mean (SD). Blinding or randomization of the groups was not performed. Data points more than two standard deviations from the mean were excluded as outliers. No statistical methods were used to estimate sample size.

## Results

### Screening of Target Molecules Specifically Expressed on Monocyte Lineage Cells

**Figure 1:**
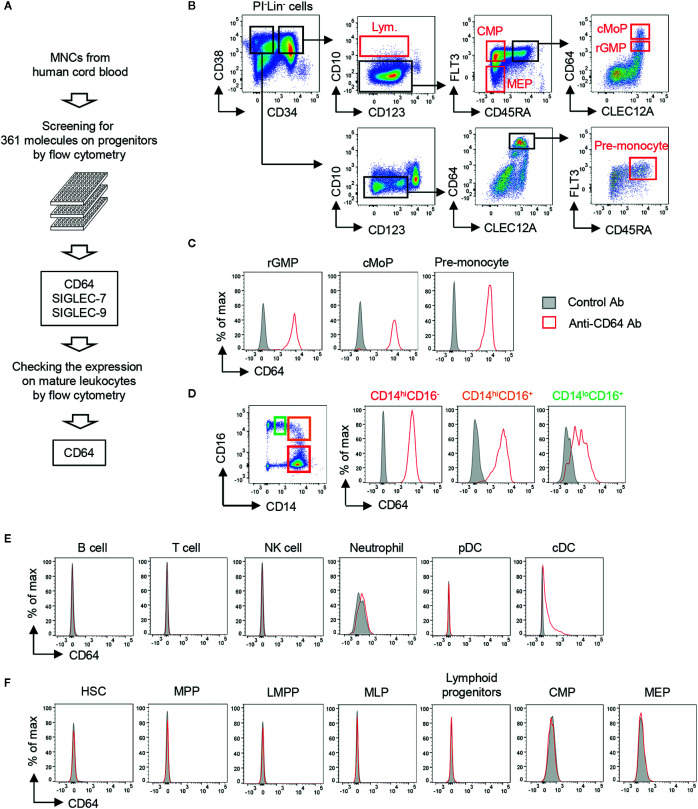
Screening of cell surface molecules to target monocytic progenitors. **(A)** Screening scheme for molecules highly restricted in monocytic progenitors. The expression of 361 cell surface molecules on HSPCs derived from UCB were examined using FCM. Expression of candidates identified from HSPC screening was further assessed on mature hematopoietic cell lineages. **(B)** Gating strategies for hematopoietic progenitors in UCB. Lineage^-^ cells (Lin^-^ cells; CD2^-^CD3^-^CD11b^-^CD14^-^CD16^-^CD19^-^CD56^-^CD235ab^-^ cells) were pre-gated. Gating strategies of other HSPCs are shown in **Supplementary Figure 1A**. **(C–F)** Expression of CD64 on mature hematopoietic cells and their progenitors. CD64 expression was evaluated on monocytic progenitors **(C)**, monocytes **(D)**, non-monocytic immune cells **(E)** and the other hematopoietic stem and progenitor cells **(F)** by FCM. Each population was identified by the following gating strategies: B cell, FSC^lo^SSC^lo^CD3^-^CD56^-^CD19^+^; T cell, FSC^lo^SSC^lo^CD3^+^; NK cell, FSC^lo^SSC^lo^CD56^+^CD3^-^; Neutrophil, SSC^hi^HLA-DR^-^CD66b^+^CD16^+^; pDC, CD3^-^CD14^-^CD19^-^CD56^-^CD11c^-^HLA-DR^+^CD123^+^; cDC, CD3^-^CD14^-^CD19^-^CD56^-^CD123^-^HLA-DR^+^CD11c^+^. Blood monocytes (HLA-DR^+^CD14^+^) were subdivided into three populations based on their expression of CD14 and CD16 as shown in (**D**, left panel). The data are representative of three independent experiments.

To target monocytic progenitors, we screened 361 cell surface molecules expressed on UCB HSPCs including revised GMP (rGMP), cMoPs and pre-monocytes (**Figures 1A, B** and **Supplementary Figure 1A**) and identified three candidates (SIGLEC-7, SIGLEC-9, and CD64) as characteristic markers of monocytic progenitors (**Figure 1C** and **Supplementary Figure 1B**). Those markers were also expressed on mature CD14^+^ monocytes (**Figure 1D** and **Supplementary Figure 1B**). However, SIGLEC-7 and SIGLEC-9 were excluded as candidates because they were also expressed on non-monocyte lineage cells, such as neutrophils, conventional dendritic cells (cDCs) and NK cells (**Supplementary Figure 1C**). On the other hand, CD64 was not expressed on most non-monocytic cells including B cells, T cells, NK cells, neutrophils and plasmacytoid dendritic cells (pDCs), although it was partially and weakly expressed on cDCs (**Figure 1E**). Importantly, CD64 expression was not observed on non-monocytic hematopoietic progenitors including hematopoietic stem cells (HSCs), multipotent progenitors (MPPs), lymphoid-primed multipotent progenitors (LMPP), multipotent lymphoid progenitors (MLPs), lymphoid progenitors, common myeloid progenitors (CMPs) and megakaryocyte-erythrocyte progenitors (MEPs) (**Figure 1F**). It has been also known that expression of *FCGR1A* encoding CD64 is not observed in non-hematopoietic cells, such as endothelial cells, epithelial cells, adipocytes and myocytes (35). Thus, CD64 is a promising marker to specifically target monocyte lineage cells.

### Generation of an ADC Targeting CD64

**Figure 2:**
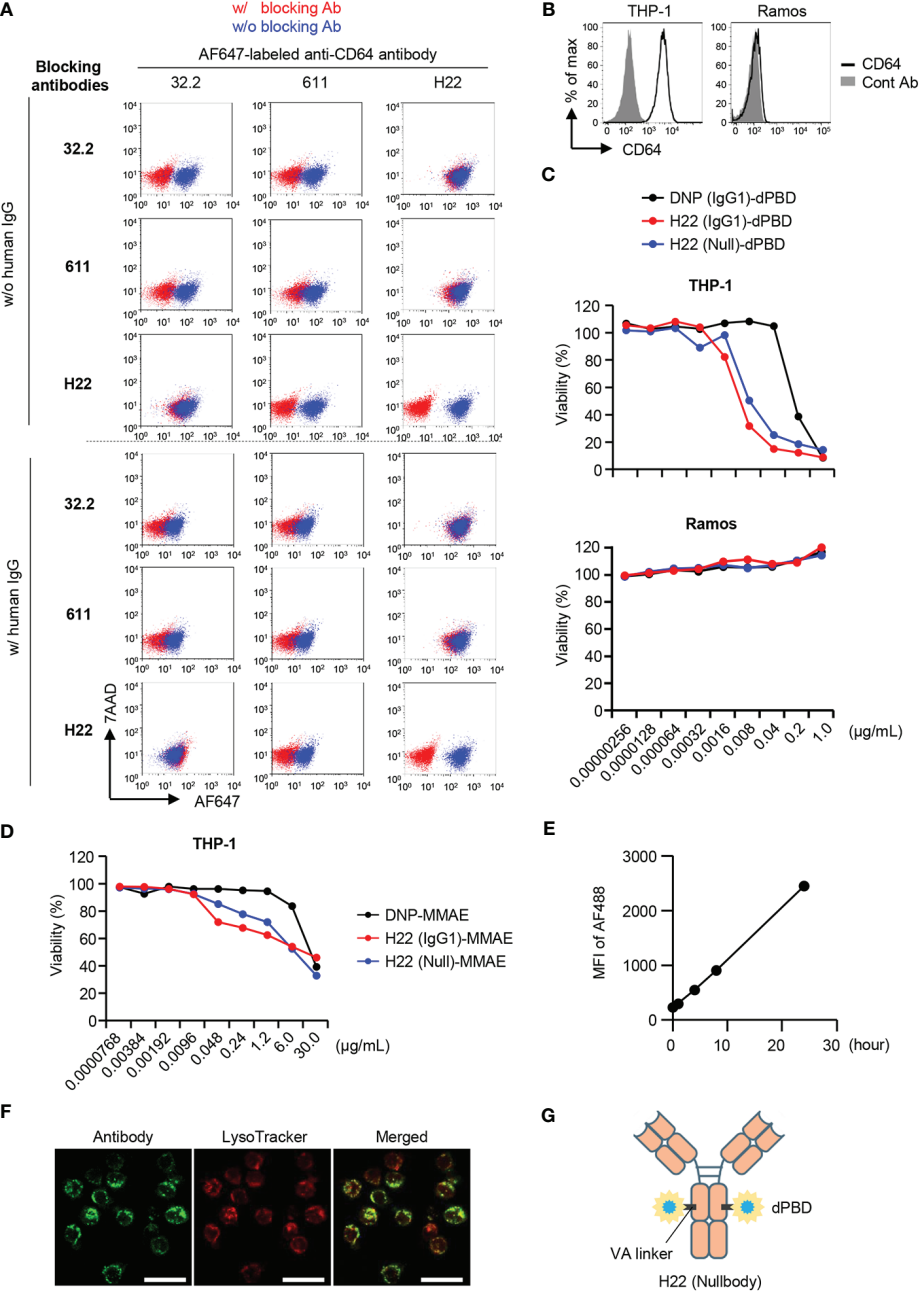
Selection of antibodies and payloads for ADC construction. **(A)** Competitive inhibition of anti-CD64 antibodies. Three clones of anti-CD64 antibodies (32.2, 611 and H22) were assessed for their capacities of epitope recognition in the presence of other clones. THP-1 cells were pretreated with blocking antibodies and then stained with Alexa Fluor 647 (AF647)-labeled anti-CD64 antibody in the presence or absence of human IgG. To estimate binding capacity, the signal intensity in the presence of the competitors (red dots) was compared with that in the absence of competitors (blue dots). **(B)** CD64 expression on THP-1 and Ramos cells. **(C)** Cytotoxicity assays with H22-dPBD. THP-1 and Ramos cells cultured with different ADCs composed of dPBD and an antibody, such as the anti-DNP antibody humanized with IgG1 (DNP(IgG1)-dPBD), H22 humanized with IgG1 (H22(IgG1)-dPBD), or H22 humanized with nullbody, an IgG4 analogue [H22(Null)-dPBD], in the presence of 20% human serum for 4 days. The relative viability of cells was estimated based on the quantification of ATP. **(D)** Cytotoxicity assay with H22-MMAE. THP-1 cells were cultured with ADCs composed of MMAE and an antibody, such as the anti-DNP antibody humanized with IgG1 [DNP(IgG1)-MMAE], H22 humanized with IgG1 (H22(IgG1)-MMAE) or H22 humanized with IgG4 nullbody (H22(Null)-MMAE), in the presence of 20% human serum for 4 days. Relative viability was estimated based on the quantification of ATP. **(E)** Internalization of H22 nullbody by U937 cells. Cells were pre-cultured with Alexa Fluor 488-labeled H22 antibody for the indicated times. The cells were then incubated with a quenching antibody on ice for 1 h and the signal intensity of Alexa Fluor 488 (AF488) was measured by FCM. **(F)** Internalization of H22 nullbody into primary monocytes. CD14^hi^CD16^-^ monocytes sorted from peripheral blood were stained with the AF647-labeled H22 nullbody. The cells were then cultured for 23 h after reaction with the H22 nullbody and stained with LysoTracker (100 nM) for 1 h. Cell images were obtained by confocal microscopy. Scale bar, 20 µm. **(G)** A schematic illustration of the structure of H22-dPBD. Data are representative of two **(A–E)** or three **(F)** independent experiments.

We next constructed an ADC against CD64 to target monocyte lineage cells, i.e., rGMP, cMoP, pre-monocytes and monocytes. To evaluate the elimination of CD64-expressing cells by the ADC, we needed another anti-CD64 Ab clone that could detect CD64 in the presence of the clone used for the ADC. We prepared three anti-CD64 Ab clones, i.e., 32.2, 611, and H22, and performed competitive inhibition assays between each clone (**Figure 2A**, upper panel). As a control, none of the clones bound CD64 after pretreatment with the same clone. Under these conditions, the recognition of CD64 by 32.2 was inhibited by pretreatment with 611 but not with H22, and 611 could not bind to CD64 when 32.2 or H22 was used as a blocking Ab. In contrast, neither 32.2 nor 611 blocked the recognition of CD64 by H22 (**Figure 2A**, upper panel). Because CD64 is an Fcγ receptor, we next tested if these clones can function in the presence of human IgG, which mimics the human serum in the body (**Figure 2A**, lower panel). Importantly, the presence of human IgG did not affect the recognition of CD64 by H22 as reported previously (36), although it somewhat decreased the CD64 recognition capacities of 32.2 and 611 (**Figure 2A**, lower panel). Based on these results, we decided to use H22 for the generation of an ADC targeting CD64 and to use 32.2 for the detection of monocytes and their progenitors.

An effective payload with cytotoxic potency and a linker are crucial components of an ADC (7). We prepared two different payloads for ADCs targeting CD64, i.e. dimeric pyrrolobenzodiazepine (dPBD), a sequence-selective DNA minor-groove binding crosslinking agent (37), and monomethyl auristatin E (MMAE), an anti-mitotic agent (38). dPBD and MMAE were linked with H22 by valine-alanine (VA) and valine-citrulline (VC), respectively, which are enzymatically cleaved in lysosomes. Cleavage of those linkers results in release of the payloads to the cytoplasm and/or the nucleus and allows them to exert their cytotoxic activities (**Supplementary Figure 2A**). We first evaluated the cytotoxic activity of H22-dPBD against THP-1 cells, a monocytic leukemia cell line expressing CD64 (**Figure 2B**). In addition to H22-dPBD humanized with IgG1 (H22(IgG1)-dPBD) and the isotype matched control ADC [DNP(IgG1)-dPBD], we also tested the nullbody H22-dPBD [H22(Null)-dPBD], in which H22 is humanized with an IgG4 analogue having three mutations that stabilize the Ab and minimize non-specific binding to Fcγ receptors (39). Compared with DNP(IgG1)-dPBD, H22(IgG1)-dPBD had a more effective level of CD64-specific cytotoxicity. For example, 0.04 µg/ml H22(IgG1)-dPBD killed most THP-1 cells, whereas the same dose of DNP(IgG1)-dPBD hardly showed any cytotoxicity against them. In addition, H22(Null)-dPBD killed THP-1 cells to the same extent as H22(IgG1)-dPBD, whereas neither H22(IgG1)-dPBD nor H22(Null)-dPBD had any cytotoxic activity against Ramos cells, a Burkitt’s lymphoma cell line that does not express CD64 (**Figures 2B, C**), suggesting that H22-dPBD functions in a CD64-specific manner. The specificity was further confirmed by competitive inhibition assay, in which pre-treatment with unconjugated H22 antibody strongly decreased the killing activity of H22-dPBD (**Supplementary Figure 2B**). Compared with H22-dPBD, the cytotoxic activity of H22-MMAE against THP-1 cells was less effective (**Figure 2D**). Finally, we confirmed that the H22 nullbody was successfully internalized by U937, a monocytic human myeloid leukemia cell line expressing CD64 (**Figure 2E** and **Supplementary Figure 2C**) and by human blood monocytes (**Figure 2F**), and that the internalized ADCs were localized in lysosomes of blood monocytes (**Figure 2F**). Based on these results, we decided to use the nullbody H22-dPBD in this study (hereafter, referred to as H22-dPBD) (**Figure 2G**).

### H22-dPBD Is Toxic for Monocytic Progenitors but Not for Monocytes

**Figure 3:**
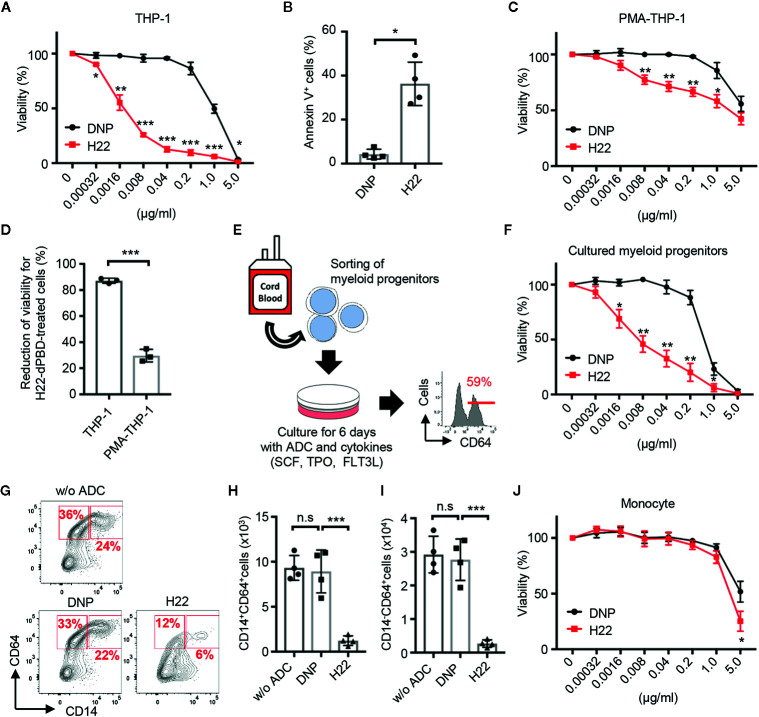
Preferential cytotoxic activity of H22-dPBD against monocytic progenitors, but not monocytes. **(A)** Evaluation of cytotoxic activity of H22-dPBD against THP-1 cells. THP-1 cells were cultured for 6 days in the presence of DNP-dPBD or H22-dPBD and their relative viability was estimated based on the quantification of ATP (n=3). **(B)** Induction of apoptosis by H22-dPBD. THP-1 cells were cultured with ADCs (0.04 µg/ml) for 3 days and the frequency of apoptotic cells was evaluated by FCM (n=4). **(C)** Killing activity of H22-dPBD against PMA-treated THP-1 cells. THP-1 cells were stimulated with 40 ng/ml PMA for 24 h prior to culture with the ADC and the susceptibility of cells to H22-dPBD was examined as shown in **(A)** (n=3). **(D)** Killing efficacy of H22-dPBD treatment against THP-1 cells and PMA-treated THP-1 cells. Normalized reduction of cell viability was calculated from the data of treatment with 0.04 μg/ml ADCs in **(A)** and **(C)** (n=3). **(E)** Experimental scheme of the killing assay against myeloid progenitors. Lin^-^CLEC12A^+^ myeloid progenitors were sorted from UCB and were cultured with ADCs and cytokines (100 ng/ml SCF, 50 ng/ml TPO and FLT3L). The number on the histogram indicates the mean frequency of CD64^+^ progenies yielded through 6-day culture (n=3). **(F)** Cytotoxic activity of H22-dPBD against cells generated from Lin^-^CLEC12A^+^ progenitors at day 6. Relative viability was assessed based on the quantification of ATP (n=3). **(G–I)** FCM analysis of CD64^+^ cells from Lin^-^CLEC12A^+^ myeloid progenitors. Cells were cultured with cytokines and 0 or 0.008 µg/ml ADC for 6 days and the numbers of CD14^+^ monocytes **(H)** and CD14^-^CD64^+^ monocytic progenitors **(I)** were determined. **(J)** Cytotoxic activity of H22-dPBD against mature monocytes. CD14^hi^CD16^-^ monocytes were sorted from the peripheral blood of healthy donors and were cultured for 6 days in the presence or absence of ADC (n=4). The data were pooled from three **(A, D)** or four **(B, J)** independent experiments or are representative of two **(F)** or three **(C, E, G–I)** independent experiments. Multiple *t*-test **(A, C, F, J),** Student’s *t*-test **(B, D)** and one-way ANOVA **(H, I)** were used to assess statistical significance. Error bars represent standard deviation of the mean. *p < 0.05, **p < 0.01, ***p < 0.001; n.s, not significant.

To observe the cytotoxic effects of ADCs, THP-1 cells were cultured for 6 days with H22-dPBD or the nullbody DNP-dPBD (hereafter DNP-dPBD) (**Figure 3A**). In this context, the viability (%) of THP-1 cells seemed to reflect cell death rather than the inhibition of cell proliferation, because the number of apoptotic THP-1 cells was significantly increased in the presence of H22-dPBD at day 3 during culture (**Figure 3B**). We next examined whether the cell cycle state affects the cytotoxic effects of H22-dPBD. THP-1 cells were treated with PMA, which induces the differentiation of THP-1 cells into macrophage-like cells with cell cycle arrest (40). Interestingly, the PMA-treatment enhanced the resistance of THP-1 cells to H22-dPBD (**Figures 3C, D**), although PMA-treated and non-treated THP-1 cells expressed CD64 at similar levels (**Supplementary Figure 2D**) and the PMA-treated THP-1 cells successfully internalized the ADC (**Supplementary Figure 2E**). Similarly, THP-1 cells that had been treated with moderate irradiation or cultured under low FBS concentrations showed less proliferation capacity (**Supplementary Figure 2F**) and inversely more resistance to the ADC treatment (**Supplementary Figure 2G**). These findings implied that H22-dPBD preferentially exerts its cytotoxic activity against proliferating cells. Thus, we further investigated the cytotoxic effects of H22-dPBD on the differentiation of primary myeloid progenitor cells into monocytes. To this end, we isolated Lin^-^CLEC12A^+^ cells from UCB, which enriched myeloid progenitor cells including CMPs, rGMPs and cMoPs (13, 41). When the cells were cultured in the presence of FLT3L, TPO and SCF for 6 days, they predominantly gave rise to CD64^+^ cells, which contains monocytes and their progenitors (**Figure 3E**). Under these conditions, the addition of H22-dPBD greatly decreased the production both of CD14^+^ monocytes and of CD64^+^CD14^-^ monocyte progenitors (**Figures 3F–I**). In contrast, H22-dPBD did not kill primary blood monocytes when added in culture (**Figure 3J**). Taken together, these results indicated that H22-dPBD selectively eliminated CD64^+^ monocyte progenitors rather than mature monocytes *in vitro*.

### Cytotoxic Effects of H22-dPBD in Humanized Mice

**Figure 4:**
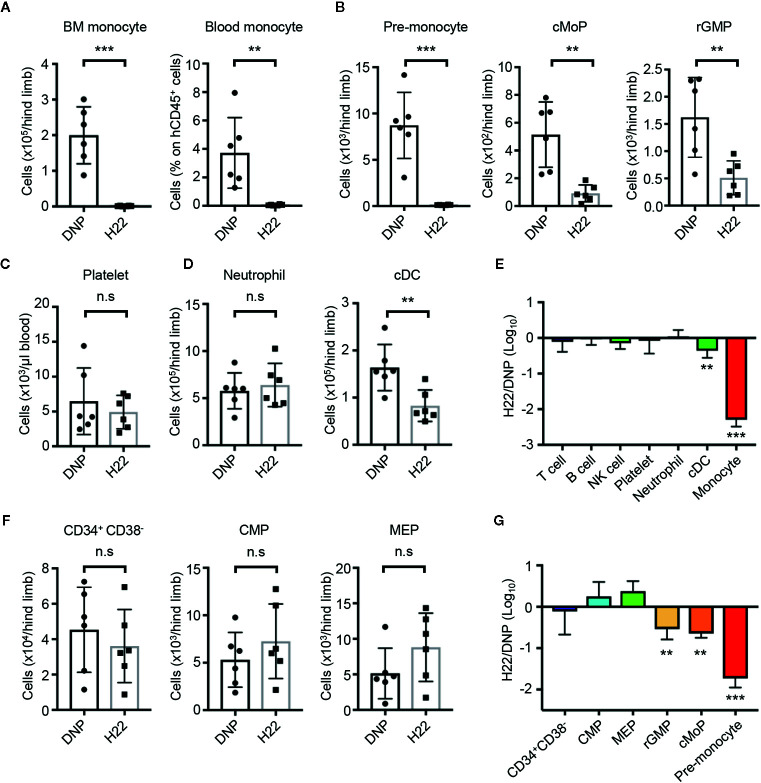
H22-dPBD-mediated elimination of monocytes and their progenitors without severe side effects in hematopoiesis. **(A–G)** BM-humanized NOG mice were generated as shown schematically in **Supplementary Figure 3**. Seven days after intravenous administration of DNP-dPBD or H22-dPBD (0.5 µg/mouse), hematopoietic cells in the BM and blood were analyzed by FCM. The number of BM monocytes and frequency of blood monocytes **(A)**, cell numbers of monocytic progenitors in the BM **(B)**, concentration of platelets (TER119^-^CD235ab^-^hCD41a^+^hCD41b^+^) in the blood **(C)**, numbers of neutrophils and cDCs in the BM **(D)**, and numbers of Lin^-^CD34^+^CD38^-^ HSPCs, CMPs, and MEPs in the BM **(F)** are shown. Ratios in cell numbers of mature immune cells in the BM and results of their statistical analyses between DNP-dPBD- and H22-dPBD-treated mice are summarized in **(E)**. Ratios in cell numbers of HSPCs and results of their statistical analyses between DNP-dPBD- and H22-dPBD-treated mice are summarized in **(G)**. Each point in the bar graphs shows the value for an individual mouse (n=6 per group). Error bars represent standard deviation of the mean. Student’s *t*-test **(A–D, F)** was used to assess statistical significance. **p < 0.01, ***p < 0.001; n.s, not significant. Data were pooled from two **(C)** or three **(A, B, D, F)** independent experiments.

Given the selective cytotoxicity of H22-dPBD against proliferating monocytic cells *in vitro*, we further examined the effects of H22-dPBD *in vivo* using humanized mice. UCB-derived Lin^-^CD34^+^ HSPCs were transplanted into sublethally irradiated *NOD/Scid/IL2Rγnull* (NOG) mice (42). Two months after the transplantation, when reconstitution of the human hematopoietic system was confirmed, 0.5 µg H22-dPBD or control DNP-dPBD was intravenously injected and the impact of ADCs was evaluated 7 days later (**Supplementary Figure 3**). As expected, the injection of H22-dPBD induced drastic reductions of monocytes in the BM and blood and their progenitors, pre-monocytes, cMoPs, and rGMPs, in the BM (**Figures 4A, B**). In contrast, there were no significant decreases in the numbers of platelets, neutrophils and lymphoid cells in H22-dPBD-treated humanized mice, although the number of cDCs was partially reduced (**Figures 4C–E**). In addition, treatment with H22-dPBD did not alter the numbers of multipotent progenitors such as Lin^-^CD34^+^CD38^-^ cells that are enriched with HSCs, CMPs and MEPs (**Figures 4F, G**). Thus, H22-dPBD selectively depleted the monocyte lineage with minimal side effects on non-monocytic lineages and multipotent progenitors *in vivo*.

### Therapeutic Effects of H22-dPBD in a PDX Model of CMML

**Figure 5:**
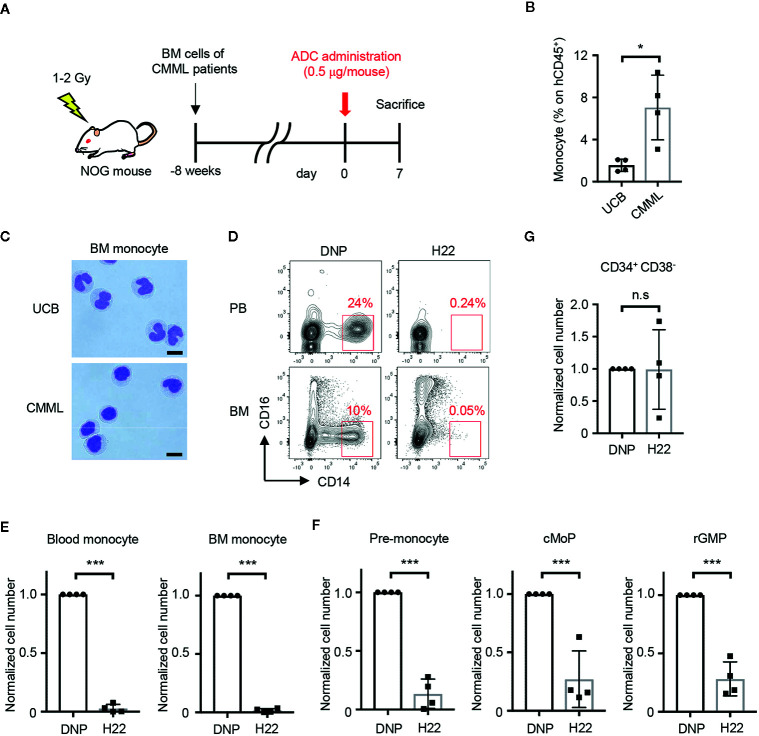
H22-dPBD eliminates patient-derived leukemic monocytes *in vivo*. **(A)** Experimental scheme showing the treatment of PDX mice with ADCs. CMML patient-derived Lin^-^CD34^+^ BM cells were transferred to sublethally irradiated NOG mice 8 weeks before the administration of DNP-dPBD or H22-dPBD (0.5 µg/mouse). The mice were analyzed 7 days after the ADC treatment. **(B, C)** Frequency of hCD45^+^ cells **(B)** and cell morphology **(C)** of BM CD14^hi^CD16^-^ monocytes obtained from NOG mice humanized with normal UCB or CMML patient-derived BM cells. Cells were sorted and stained with Diff-quik (scale bars, 5 µm). **(D)** Representative FCM plots of hCD45^+^ cells in the peripheral blood (upper panels) or BM (lower panels) from DNP-dPBD- or H22-dPBD-treated PDX mice. **(E–G)** Impact of ADC-treatment in hematopoiesis of PDX mice. Cell numbers of monocytes, monocytic progenitors and Lin^-^CD34^+^CD38^-^ cells are shown in **(E)**, **(F)**, and **(G)**, respectively. Each point in the bar graphs shows the value for an individual mouse (n=4 per group). Error bars represent standard deviation of the mean. Student’s *t*-test was used to assess statistical significance. *p < 0.05, ***p < 0.001; n.s, not significant. Data are representative **(C, D)** or pooled **(B, E–G)** from four independent experiments.

The selective monocyte-removing effect of H22-dPBD *in vivo* was reminiscent of its application to CMML, a type of leukemia with increased numbers of monocytes and immature blood cells. To prepare PDX mice, BM cells obtained from CMML patients were transplanted into sublethally irradiated NOG mice (**Figure 5A**, **Supplementary Table 1**). Upon reconstitution, the frequency of monocytes in human CD45^+^ cells of CMML PDX mice was much higher than that of NOG mice humanized with UCB cells (**Figure 5B**). In addition, the monocytes in the PDX mice showed the characteristic morphology of monocytic dysplasia such as round nuclei and high nuclear-cytoplasmic ratios (**Figure 5C**, lower panel), in contrast to the horseshoe-shaped nuclei of UCB-derived normal monocytes (**Figure 5C**, upper panel). Thus, the pathology of CMML was recapitulated in the PDX mice.

To test the therapeutic effects of H22-dPBD, H22-dPBD or control DNP-dPBD was administered once into the PDX mice and the efficacy of leukemic monocyte removal was evaluated on day 7 (**Figure 5A**). Importantly, both the CD14^hi^ leukemic monocytes and the monocyte progenitors were almost completely depleted in the blood and/or BM of H22-dPBD-treated CMML PDX mice (**Figures 5D–F**, **Supplementary Figures 4A, B**). In contrast, there was no significant difference in the number of Lin^-^CD34^+^CD38^-^ HSPCs between those two groups (**Figure 5G**, **Supplementary Figure 4C**). Thus, H22-dPBD could successfully remove leukemic monocytes without severe side effects on hematopoiesis.

We then attempted to evaluate the impact of H22-dPBD treatment on the overall survival of CMML PDX mice. However, those mice did not effectively support the engraftment of HSPCs derived from CMML patients, and with the low chimerism, it was impossible to test their survival. Therefore, we transplanted THP-1 cells to sublethally irradiated NOG mice and treated the mice with H22-dPBD or DNP-dPBD at 2 and at 4 weeks after the xenografting (**Supplementary Figure 5A**). Following the injection if H22-dPBD, THP-1 cells were dramatically eliminated in the BM (**Supplementary Figure 5B**) and the survival rate of xenografted mice was significantly prolonged (**Supplementary Figure 5C**). These results suggested the usefulness of H22-dPBD as a therapeutic agent for CMML patients.

### H22-dPBD Prevents Solid Tumor Development by Eliminating TAMs

**Figure 6:**
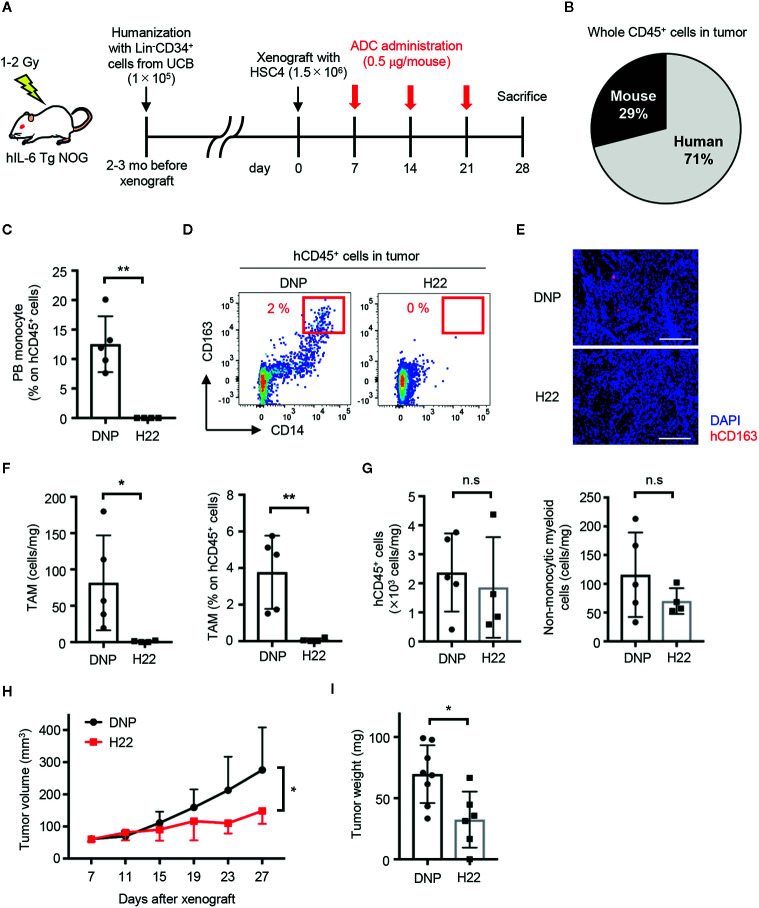
H22-dPBD eliminates TAMs and inhibits the development of solid tumors. **(A)** Experimental scheme of the generation and therapeutic treatment of solid tumor-bearing humanized mice with ADCs. At day 0, HSC4 cells (1.5×10^6^ cells/mouse) were subcutaneously transplanted in hIL-6 Tg NOG mice humanized with UCB-derived Lin^-^CD34^+^ cells. DNP- or H22-dPBD (0.5 µg/mouse) were intravenously injected to the mice once a week from day 7 and tumor sizes were measured every 4 days from day 7. The mice were sacrificed at day 28 and tumor weights and cells infiltrating the tumors were evaluated. mo: months. **(B)** Ratio of hCD45^+^ and mCD45^+^ cells in total leukocytes in the tumor. **(C)** Frequency of monocytes in circulating hCD45^+^ cells at day 28. **(D)** Representative FCM plots of tumor-infiltrating hCD45^+^ cells. Frequencies of CD163^hi^CD14^+^ TAM-like cells are shown. **(E)** Histological evaluation of tumor-infiltrating CD163^+^ cells in DNP-dPBD- or H22-dPBD-treated mice. Scale bars: 200 μm. **(F)** Number and frequency of TAMs in DNP-dPBD- or H22-dPBD-treated mice. **(G)** Numbers of total hCD45^+^ cells and hCD45^+^CD3^-^CD19^-^CD56^-^CD14^-^ (non-lymphoid, non-monocytic) cells in the tumor. **(H)** Time-course analysis of tumor development. Tumor volumes were calculated according to the following formula; 0.5 × length × (width)^2^. Statistical analysis was performed on data at day 27. Averages of tumor size in each group were shown. **(I)** Tumor weights of DNP-dPBD- or H22-dPBD-treated mice at day 28. Error bars represent standard deviation of the mean. Each point in the bar graphs shows the value for an individual mouse (DNP-treated group: n=8, H22-treated group: n=6). Student’s *t*-test was performed to assess statistical significance. *p < 0.05, **p < 0.01; n.s, not significant. Data are representative of three independent experiments **(D, E)** or are pooled from two independent experiments **(B, C, F–I)**. Data points more than two standard deviations from the mean were excluded as outliers **(H, I)**.

Because TAMs can be derived from monocytes (18, 20), it was worth investigating whether treatment with H22-dPBD can eliminate TAMs through the depletion of their progenitors and would lead to the reduction of solid tumor size. According to a previous report, we transplanted Lin^-^CD34^+^ UCB cells into human IL-6-Tg NOG mice, which generates TAMs with a functionally immunosuppressive nature and show an enhanced tumor-growth (29). A few months later, we subcutaneously transplanted HSC4 human squamous carcinoma cells into those humanized mice (**Figure 6A**). In this context, HSC4 cells did not express CD64 and thus were not targeted by H22-dPBD treatment *in vitro* (**Supplementary Figure 6**). Before injecting H22-dPBD, we confirmed that the chimerism of hCD45^+^ cells and the frequency of monocytes in hCD45^+^ cells were similar between the H22-dPBD- and DNP-dPBD-treatment groups (data not shown). Additionally, hCD45^+^ cells were dominant in tumor-infiltrating leukocytes in this model (**Figure 6B**). Under these conditions, treatment with H22-dPBD effectively eradicated both blood monocytes and tumor-infiltrating CD14^+^ cells, some of which expressed CD163, a representative TAM marker (43, 44) (**Figures 6C–F**). In contrast, the numbers of total human leukocytes and non-monocytic myeloid cells in the tumor were unaffected (**Figure 6G**), suggesting there were minimal side effects in tumor-infiltrated non-monocytic cell lineages. Importantly, we found that the HSC4 tumor development was significantly suppressed in both volume and weight at 3 weeks after the initial treatment with H22-dPBD (**Figures 6H, I**). Collectively, these results demonstrate that anti-CD64 dPBD is a promising agent to directly treat monocytic leukemia and indirectly suppress solid tumor development through the depletion of TAMs.

## Discussion

As cytotoxic side effects caused by anti-cancer drug treatments are major challenges for the treatment of leukemias, various drug-delivery systems including ADCs have been developed to specifically target cancer cells. In this context, the H22-dPBD ADC that we generated in this study has a unique characteristic. Although the H22 nullbody binds both to monocyte progenitors and to mature monocytes, the H22-dPBD ADC selectively eliminated proliferating monocyte progenitors. This could be because the DNA-crosslinking caused by dPBD is more cytotoxic for proliferating cells, in which replication and transcription of DNA are actively occurring. Supporting this notion, the inhibition of THP-1 cell proliferation by treatment with PMA clearly decreased the sensitivity against H22-dPBD, although it did not alter the level of CD64 expression or ADC internalization. Given the difference in proliferation potential between cell lines and primary cells, at least in some cases, cell line-based studies may not reflect the true therapeutic efficacy and target cells of drugs, which can lead to misinterpretation.

Because CD64 is a well-known marker for monocytes and macrophages (36, 45), various ADCs against CD64 have been generated to eliminate leukemic monocytes and/or inflammatory macrophages (46–51). However, in most cases, the efficacy of ADCs was tested in culture and/or in a xenograft model using leukemic cell lines. Thus, it remained unclear if anti-CD64 ADCs work to remove patient-derived monocytic leukemia cells *in vivo*. In addition, the side effects of anti-CD64 ADCs on other cell lineages, especially hematopoietic progenitors, have never been evaluated to date. However, our recent findings, i.e., the identification of CD64^+^ monocytic progenitors, such as rGMP, cMoP and pre-monocytes, provided an opportunity to reconsider and review the utility of anti-CD64 ADC for anti-cancer therapy and side effects on hematopoiesis.

Under that background, we developed a new ADC targeting human CD64 (H22-dPBD), which kills monocytic progenitors, but not monocytes or other progenitors, and demonstrated that targeting monocytic progenitors with that ADC sharply decreased the number of monocytes in the BM and blood of humanized mice. Treatment with H22-dPBD partially reduced the number of cDCs, which might reflect the presence of monocyte-derived cDCs among total cDCs. Interestingly, treatment with H22-dPBD did not alter the number of neutrophils in humanized mice. This was unexpected because treatment with H22-dPBD *in vivo* partially decreased rGMPs that have the potential to give rise to granulocytes and monocytes. Since neutrophils are short-lived cells, this result may reflect an alternative differentiation pathway of neutrophil generation through unknown progenitors. It might also be possible that H22-dPBD preferentially eliminated monocyte-committed rGMPs, but not neutrophil-committed rGMPs. Indeed, both in mice and in humans, a neutrophil-committed progenitor was identified in the GMP fraction (52, 53). Another possible explanation is the mechanism that stabilizes neutrophil production such as a neutrophil rheostat (neutrostat) (54–56). Given that neutrophils are short-lived cells that first appear in an emergency, there might be complicated regulatory mechanisms of neutrophil homeostasis *in vivo*.

It has been suggested that TAMs are resistant to anti-CD64 ADCs because of their lower expression level of CD64 and the higher degradation capacity of protein-based anti-cancer drugs than are M1 macrophages (28, 36, 37). Considering the property of H22-dPBD to effectively kill monocytic progenitors, it can eradicate any monocyte-derived cells regardless of their nature such as their proliferation state, their capacities to degrade and effuse drugs, and their expression level of CD64. Thus, anti-CD64 ADCs are suitable agents that can target highly heterogeneous monocyte-derived cells such as TAMs. Indeed, in our solid tumor-bearing humanized mouse model, tumor-infiltrating CD163^+^ TAM-like cells were successfully removed by treatment with H22-dPBD. Importantly, the depletion of TAMs by H22-dPBD significantly attenuated solid tumor progression *in vivo*, suggesting the utility of anti-CD64 ADCs for anti-solid tumor therapy. In this study, we administered ADCs three times in the observation period, because it takes a long time for solid tumors to progress. Although those treatments did not significantly alter the numbers of hCD45^+^ cells and non-monocytic myeloid cells that infiltrated in the tumor, we noticed that the number of BM neutrophils was reduced (data not shown). This might be due to the reduction of rGMP by three injections of H22-dPBD, which could be stronger than a single injection. However, owing to the properties of the humanized mouse model, in which neutrophils poorly egress from the BM and circulate in the peripheral blood (29), the attenuation of tumor development was probably not due to the reduction of neutrophils in this model. To minimize the side effects of the three injections on BM neutrophils, further optimization of dosages and injection intervals will be required.

Tissue-resident macrophages are derived either from yolk sac progenitors during the embryonic stage or from monocytes (57). In this study, we successfully eliminated TAMs generated from human monocytes in humanized hIL-6-Tg NOG mice. On the other hand, TAMs from yolk sac-derived macrophages cannot be evaluated in tumor-bearing humanized mouse models, suggesting their limitation and a requirement for further technological innovation.

In summary, H22-dPBD is a unique ADC that selectively targets proliferating monocyte-committed progenitors and is effective in treating monocytic leukemia and TAM-targeted suppression of solid tumor development (**Supplementary Figure 7**). Since monocytes and monocyte-derived cells including macrophages, DCs and osteoclasts play key roles in the progression of various pathological conditions (28), the strategy targeting monocytic progenitors might also be applicable to treating a variety of other disorders, in particular those involving excessive inflammation and autoimmunity.

## Data Availability Statement

The raw data supporting the conclusions of this article will be made available by the authors, without undue reservation.

## Ethics Statement

The studies involving human participants were reviewed and approved by the institution ethical committee of Tokyo Medical and Dental University and Yokosuka Kyosai Hospital. The patients/participants provided their written informed consent to participate in this study. The animal study was reviewed and approved by the Institutional Animal Care Committee of the Tokyo Medical and Dental University.

## Author Contributions

YI and MKan planned and performed the majority of experiments. MKai and ZS generated ADCs and evaluated activities with *in vitro* experiments. SK performed the screening of molecules expressed on monocytic progenitors. MA, MY, TN, KO, NK, and ST provided samples from patients and YI, MKan, and TO conceived of the project and wrote the manuscript. All authors contributed to the article and approved the submitted version.

## Funding

This research was supported by the AMED (#JP19cm0106333, TO).

## Conflict of Interest

MKai and ZS are employees of Kyowa Kirin Co., Ltd.

The remaining authors declare that the research was conducted in the absence of any commercial or financial relationships that could be construed as a potential conflict of interest.
