# Supplementary material for: An Antibody-Drug Conjugate That Selectively Targets Human Monocyte Progenitors for Anti-Cancer Therapy
Source: Front Immunol. 2021 Feb 22;12:618081. doi: 10.3389/fimmu.2021.618081 (PMC7937628; doi:10.3389/fimmu.2021.618081)
Supplement: Supplementary file 8 [file Table_1.pdf]

Supplementary Table 1 | Each BM sample was transplanted into two mice, which were for H22-dPBD and DNP-dPBD-treatment. Chimerisms in PB and BM of DNP-dPBD-treated group were examined before and 1 week after ADC-treatment, respectively. PDX mice derived from P#1 and P#4-6 patients were used for the experiments in Figure 5.

Patient						PDX mouse		
ID	Age	Sex	Diagnosis	Karyotype	Treatment	PB chimerism	BM chimerism	Engraftment
P#1	77	M	CMML-2	normal	no	5.4%	49.4%	yes
P#2	79	F	CMML-2	normal	no	1.7%	48.5%	yes
P#3	67	M	CMML-2	47, XY, +8[18]/46, XY[2]	no	0.0%	0.2%	no
P#4	79	M	CMML-2	normal	no	2.3%	32.7%	yes
P#5	73	F	CMML-0	undeterminable	no	0.3%	11.6%	yes
P#6	88	M	CMML-1	normal	no	4.3%	56.3%	yes
